# On Excretion

**Published:** 1905-01-21

**Authors:** Stuart Tidey


### HOSPITAL CLINICS.

### ON EXCRETION.

By STUART TIDEY, M.D.

"Not that which goeth into the man, but that which goeth out of the man, defileth the man."

THE doctrine of auto-infection needs enforcing as eloquently to-day as it did two thousand years ago. The manufacture of anti-constipation pills flourishes in spite of the "open door," while every second highly civilised person needs artificial aid to perform a natural function.

In order to maintain healthy combustion, the supply of fuel to the vital furnace needs supplementing by the systematic removal of cinders and ashes—the former through the intestines, the latter through the kidneys. The present letter concerns intestinal evacuation.

The ideal is the daily evacuation—mark the word, no perfunctory motion, but effective evacuation—short of this constipation results, a condition the gradations of which vary in number to infinity. There may be the daily motion, which eliminates not the waste matter of the preceding day, but of the day, or two days before that; or the daily motion may represent only a portion of the amount of daily refuse, in which case the excess accumulates until the occasional purge clears the way for a fresh accumulation to form; or there is only a discharge at longer intervals, and a real evacuation becomes a rare phenomenon.

The chronic poisoning which results in all these stages of constipation gives rise to innumerable symptoms, more or less grave, and the sufferer comes to regard them as essential constitutional peculiarities, with which it is useless to contend save by the periodic pill or the occasional morning saline.

Apart from the chronic constitutional results of constipation, such as headaches, foul breath, digestive disturbances, anæmia, apoplexy, and insanity, I am of opinion that it is a most important factor in the etiology of the acute specific fevers, such as scarlatina, measles, diphtheria, and typhoid. In the first set of cases the effect results direct by chronic blood and tissue poisoning; in the second set, by reducing the resisting power of mucous membranes, and protracting the sojourn of specific poisons in contact with absorbent surfaces.

Realising the important part that constipation plays in the etiology of disease, it becomes a question of the utmost importance how to combat a condition which is so widely prevalent. How can we ensure the daily ideal evacuation? It is a question of action and reaction between the bowel and its contents. The contents must be effectively stimulating to the bowel, and the bowel must be effectually responsive. The stimulating properties of the contents will depend partly upon bulk and partly on quality. In order to obtain adequate bulk, the food taken should contain a considerable proportion of indigestible matter, so as to leave a sufficient residue. The fibrous and granular nature of the residue will serve to stimulate the mucous membrane to secrete and lubricate its surface.

In order to stimulate the healthy hollow organ to contract and expel its contents, the contained substance must be of sufficient bulk to put the neuromuscular walls on the stretch. The physical stimulus then engendered will lead to the appropriate expulsive contraction, whether it be the rhythmical contraction of the heart, the periodic contraction of the uterus, the automatic volitional contraction of the bladder or the vermiform contraction of the bowel. The patient who cannot swallow a pill because it is too large constantly swallows boluses of food many times larger. The pill is really too small until, supplemented by the mouthful of water, the combined mass acquires sufficient bulk to be grasped by the pharyngeal constrictors and be passed into the œsophagus.

In like manner, no self-respecting bowel will contract on the insignificant refuse of a highly concentrated meal. There is no stimulus, so the bowel remains inactive, and by repetition the inactivity becomes habitual and leads to loss of contractile power. The highly nitrogenous refuse affords a nidus for bacillus coli and his congeners; it undergoes fermentation, generates gases which distend the bowel, and a condition of chronic flatulence ensues.

The food, then, should be of such a kind as to supply a sufficient residue to stimulate the vermiform contraction of the bowel. To promote a like object, the interval between meals should be adequate, as small, frequent meals tend, among their disadvantages, to destroy the appetite, and so to hinder the consumption of a sufficient bulk of food to stimulate effective contraction of the bowel.

The residue should contain substances which in themselves serve to stimulate contraction of the bowel, such as bran, husk of grain, skins of fruit, pips carefully chewed, and vegetable and meat fibre.

The wholesale swallowing of cherry stones, and the popular use, as an aperient, of whole linseed mixed with warm water, and taken as a drink, both of which are practised in parts of France, no doubt act as effectual stimulants of the bowel, and seem devoid of special risk.

A draught of water three or four times daily, half an hour or so before meals, so as to enter an empty stomach, is a great aid towards securing the daily evacuation. The idea that the consumption of water tends to make fat may be relegated to the refuse heap of popular delusions.

To turn from the subject of nutrimental residue and water as essential aids to the natural performance of the intestinal functions, I will now endeavour to show how the intestine may be kept in a state of functional alertness so as to respond readily to the stimulus of its contents. In the first place, a habit of evacuating the bowels daily at the same hour should be cultivated. Childhood is the period for initiating this habit: "Train up a child in the way he should go; and when he is old, he will not depart from it." More important is this than every other canon of health: more important than feeding, more important than education. The daily evacuation means health and happiness; the absence of it means a departure from health and an endless train of physical and moral backsliding. The naughty child is one with a loaded bowel and a congested liver, or else his nurse is affected that way; a dose of calomel administered to the right person will make him good. The lady who kept a box of pills for family use, of which she gave one to any individual member who was out of temper, and when they were all out of temper, took one herself, had a correct appreciation of the evils of auto-infection. In childhood a proper quality of food, given at suitable intervals, and the initiation of a habit, are the chief factors concerned in procuring the daily evacuation.

As time goes on and life becomes more complicated, other factors come into play. The circumstances of school life, involving sedentary occupations, sometimes conducted in ill-ventilated rooms, sometimes associated with shortage of closet accommodation, or absence of opportunity, tend to interfere with the regular pursuit of the habit. The schoolmaster should provide one closet for every six boys, and see that each boy paid it a daily visit, with a proper result.

Girls appear to be more careless than boys in this matter, and as they grow up they are exposed to a special danger. I refer to the wearing of a supporting garment around the abdomen and back, leaving aside for the moment all question of abdominal compression by tight lacing.

Now this supporting garment is worn for a double purpose, namely, to give attachment to the skirts and lower garments, and to support the body. With reference to the first purpose, which does not directly concern the present question, I shall simply suggest that it is far better to banish the meridianal supporting appliance and wear woven under garments fitting the body like a skin, and of a thickness suitable to existing meteorological conditions, while the outer garments should hang from the shoulders, and merely be caught in at the waist for convenience. As a support to the body the corset has very serious disadvantages, and none is more serious than that which concerns its effect on the excretory functions of the bowel.

I said above that in order to insure the contractility of the bowel two conditions are necessary, viz., an adequate stimulus depending on the bulk and nature of the food residue, and an adequate responsive tonicity of the muscles involved. Muscular tone depends partly on the condition of the muscle fibres themselves. In order to maintain the muscle fibres in a good working condition, they must be regularly exercised. When allowed to remain inactive they undergo degeneration, and become weak and yielding. Now the muscles concerned in intestinal excretion are of two kinds; first, the involuntary muscles in the intestinal walls, the tone of which maintains the bowel in a state of semi-contraction, and the vermiform contraction of which conveys the intestinal contents onward; and the voluntary muscles occupying the abdominal walls, the tone of which maintains the form of the abdomen, and the voluntary contraction of which aids in the expulsion of the intestinal contents.

Should either of these sets of muscles lose tone or contractile vigour, the bowel and abdominal walls will tend to yield to intra-abdominal expansile force. This force is being constantly generated in the form of fermentation gases in the bowel. Their volume depends in a great measure on the length of sojourn in the bowel of food residue, and the exigencies of modern life frequently necessitate their voluntary retention when they would naturally escape.

The condition of things, therefore, is a balance of power between expansile intestinal gases and muscular tonicity. So long as the muscle tone is good, it will resist the expansile force, and the normal shape of the figure will be maintained; but as the muscles lose tone, the gases overcome them and the abdomen becomes distended. Prominent among the many causes which lead to loss of muscular tone is a lack of demand on their contractility, a relegation of their function, as it were, to an outsider. This is precisely what happens when an abdominal support is worn. The support supplants the muscles in their function of withstanding the expansile force of the intra-intestinal gases, and they consequently lose tone and contractile vigour, while a condition of progressively increasing abdominal girth is brought about. This condition is most marked in women who have passed the meridian of life, but its origin dates from the first wearing of the abdominal support. The evil of the abdominal support does not end here; apart from supplanting the intestinal and abdominal muscles in their function, it also hinders freedom of movement, so that the muscles on which the maintenance of the figure depends undergo degenerative changes, and become weak and flabby through lack of exercise. The preservation of the figure and of health both, therefore, depend on the development and tone of the intestinal and abdominal muscles, which can be secured only by their functional use and free exercise. This object demands the abolition of all artificial supporting bands, and the daily practice of systematic exercises, or the pursuit of a physically active life.

Systematic movements to develop the waist muscles may be practised with or without artificial aids. Deep abdominal breathing is a valuable means of developing the abdominal muscles, and one of the simplest means of practising it is to lie on the ground, place a considerable weight on the abdomen, and make it go up and down by means of deep abdominal breathing. The kind of exercise is of less importance than the regularity with which it is practised.

As an erst pathologist as well as present clinical observer, I will say a few words on the evils of tight lacing. Over and above the disadvantages quâ support, tight lacing produces a train of physiological defects and physical deformities. It hampers the movements of the intestines, checks the play of the chest walls where they should be most freely moveable, thrusts the abdominal organs upwards so as to encroach on the thoracic space, thereby impeding respiration and the heart's action, and dislocates the normal distribution of blood, of which evidence is sometimes afforded by a feeling of faintness occurring when the lacing is loosened. Besides these instances of dislocation, tight lacing cuts into abdominal organs, and may lead to actual amputation of a portion of the liver.

To recapitulate the natural means of securing a daily intestinal evacuation :—

1. The meals should not exceed three a day.
2. Food should contain a considerable bulk of indigestible matter, and be well masticated.

3. A good drink of water should be taken half an hour before each meal.
4. A habit of daily evacuation at a certain hour should be inculcated.
5. There should be no support of the abdominal walls, and no restraint of muscular action.
6. The waist muscles should be regularly exercised, either by systematic movements, the pursuit of sport, or of regular physical work.

